# Supplementary material for: Weak effects of local prey density and spatial overlap on predation intensity in a temperate marine ecosystem
Source: Ecol Appl. 2025 Nov 21;35(7):e70136. doi: 10.1002/eap.70136 (PMC12638530; doi:10.1002/eap.70136)
Supplement: Supplementary file 1 — Appendix S1. [file EAP-35-e70136-s001.pdf]

Appendix S1

Ecological Applications

Title: Weak effects of local prey density and spatial overlap on predation intensity in a temperate marine ecosystem

Authors: Max Lindmark, Christopher A. Griffiths, Valerio Bartolino, Viktor Thunell, Federico Maioli, Sean C. Anderson, Andrea Belgrano, Michele Casini, Katarzyna Nadolna-Ałtyn, Joanna Pawlak, Marzenna Pachur, Marcin Rakowski, Karolina Wikström, Murray S. A. Thompson, Mayya Gogina, Didzis Ustups, Nis S. Jacobsen

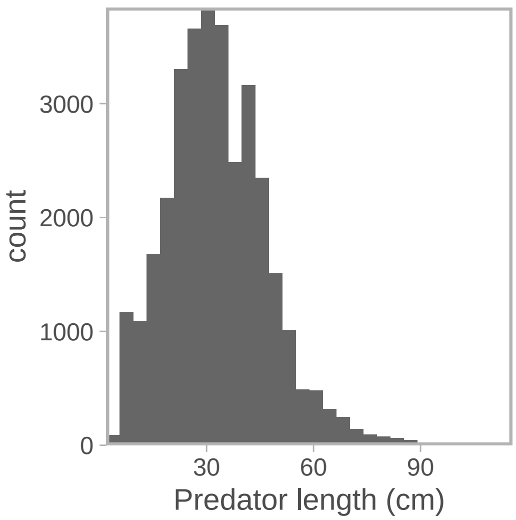

Figure S1: Distribution of cod sizes in the stomach content data.

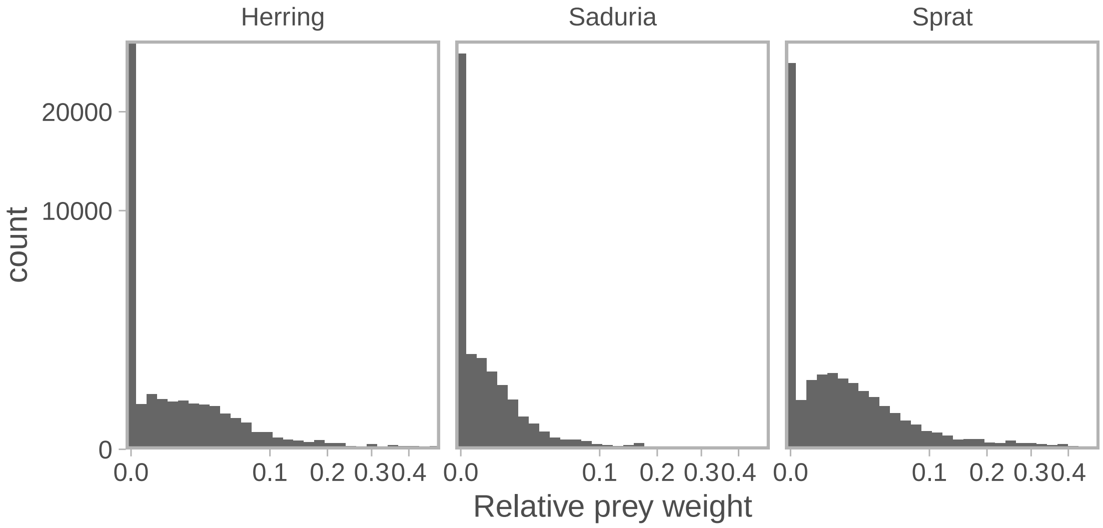

Figure S2: Distribution of relative prey weights. Note the axes are square-root transformed to better view the distribution.

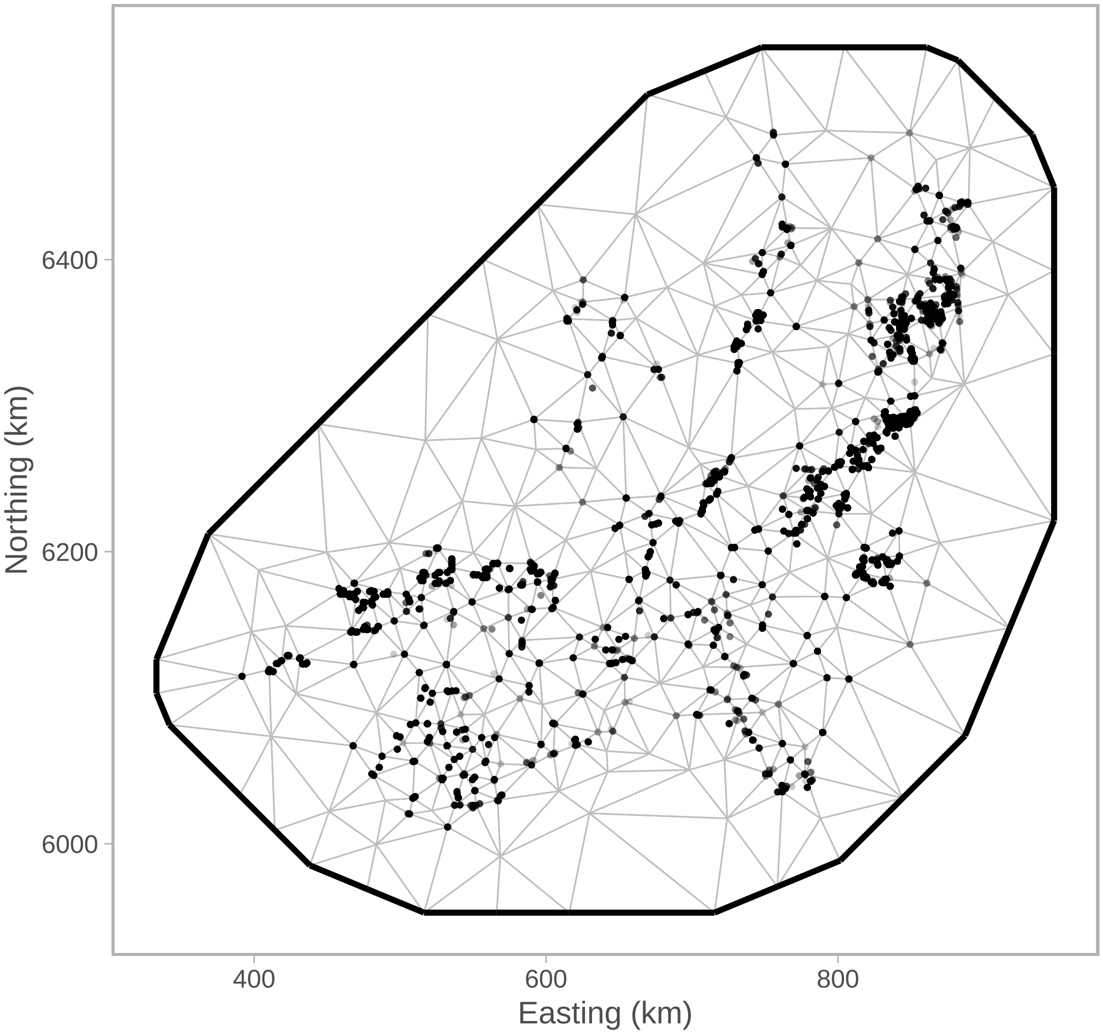

Figure S3: SPDE mesh for the stomach content models.

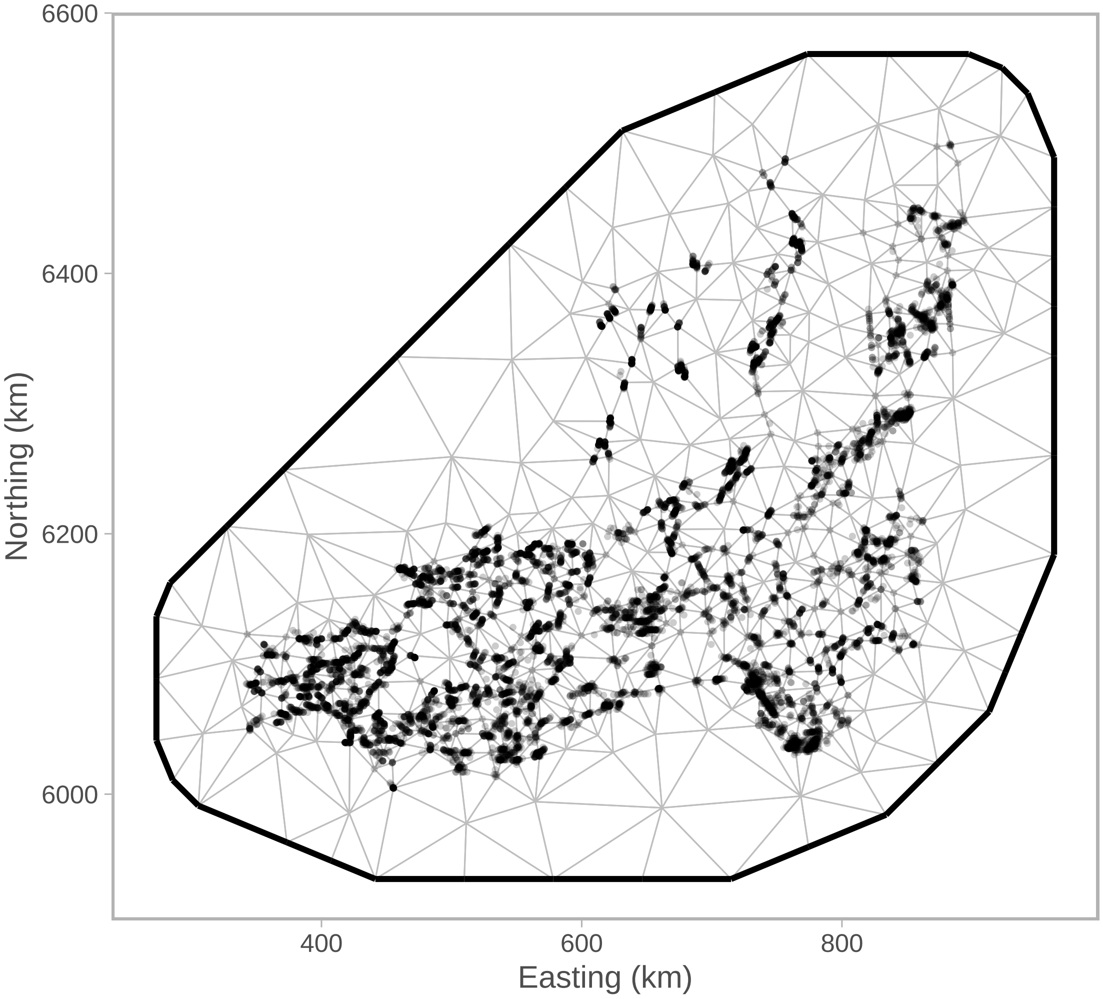

Figure S4: SPDE mesh for the biomass density model.

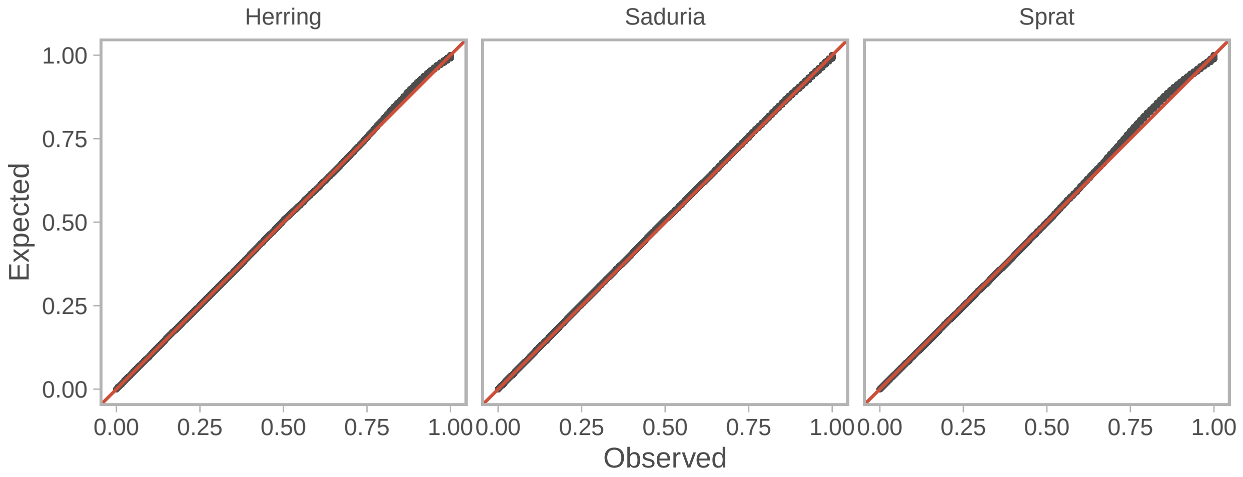

Figure S5: QQ-plots of the stomach content models based on simulated randomized quantile residuals (Dunn *et al*. 1996, Hartig 2022).

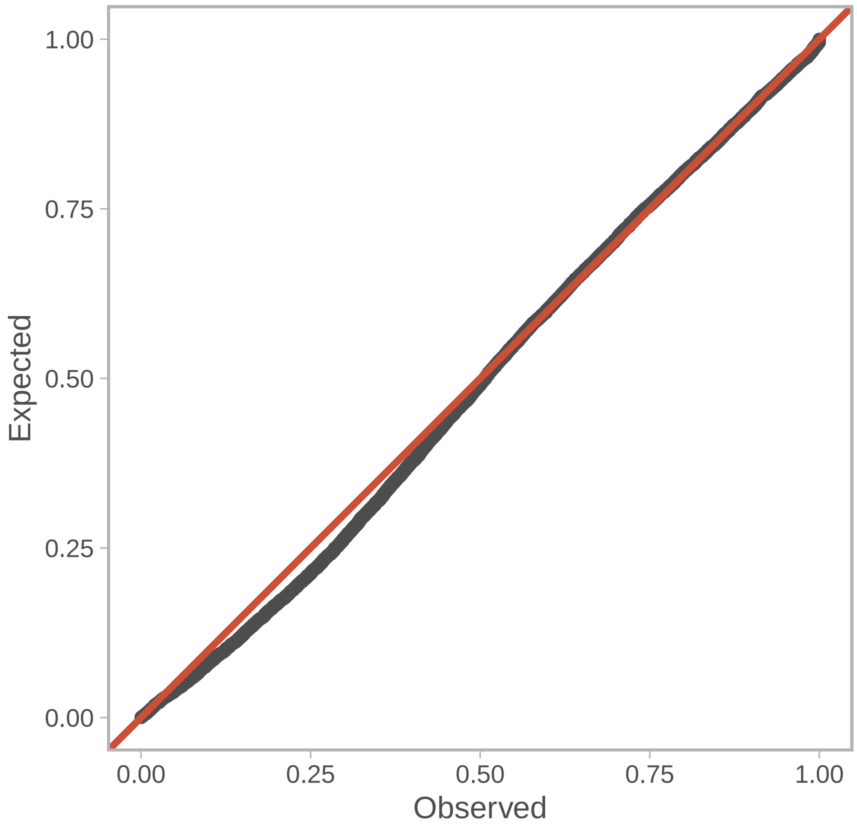

Figure S6: QQ-plots of the cod biomass density model based on simulated randomized quantile residuals (Dunn *et al*. 1996, Hartig 2022).

Table S1: Estimates for the prey-effects on the link scale (log for both linear predictors) for *Saduria*.

Model	Coefficient	Estimate	Standard error
Binomial	Slope	1.28	0.19
Binomial	Breakpoint	-0.29	0.08
Gamma	Slope	0.25	0.17
Gamma	Breakpoint	-0.87	0.36

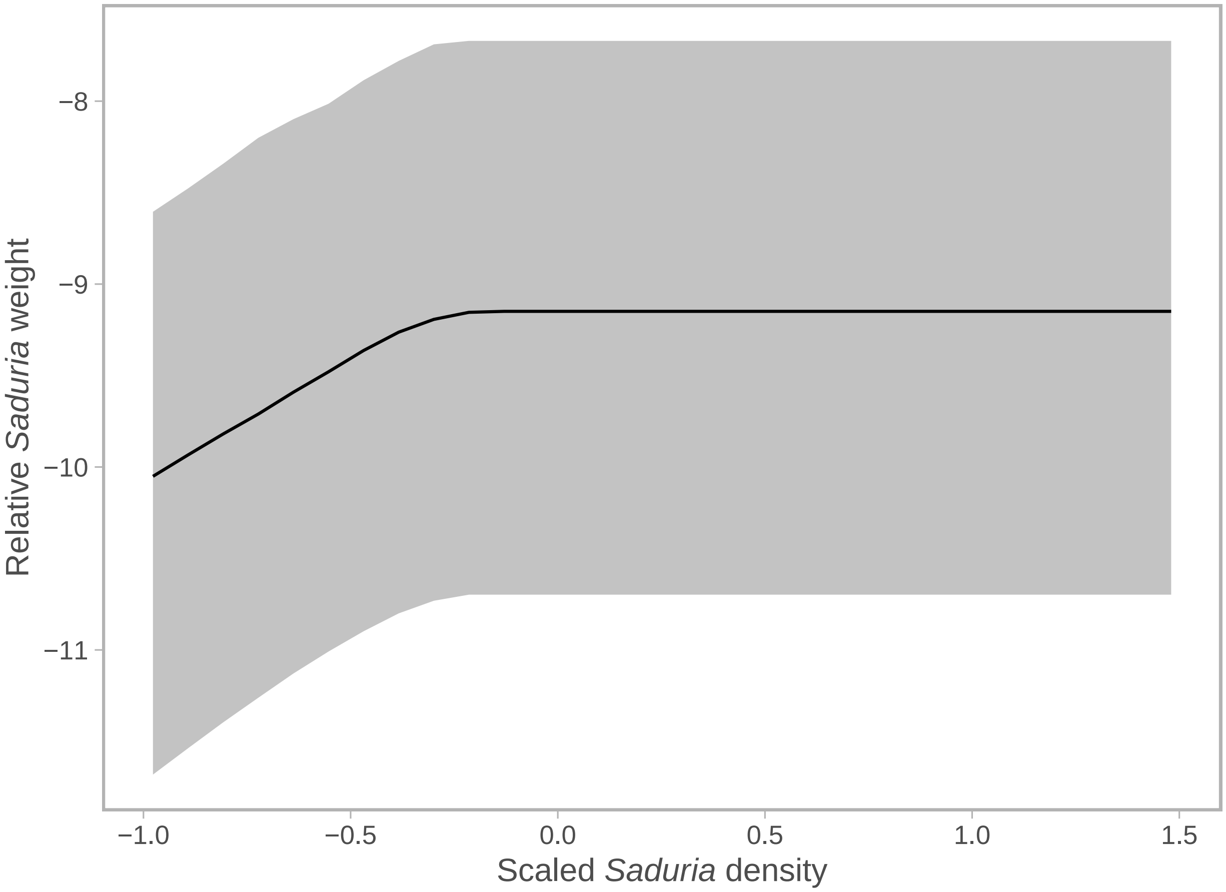

Figure S7: Conditional effects of *Saduria* biomass density for the relative prey weight of *Saduria*. The prediction (on the link scale) is for the total relative prey weight, i.e. both model components. The solid line depicts the median, and the shaded area covers the 10th–90th percentile of 500 draws from the joint precision matrix. The prediction is done with random effects omitted, for a cod of mean length (33 cm), and at the mean depth in 2019.

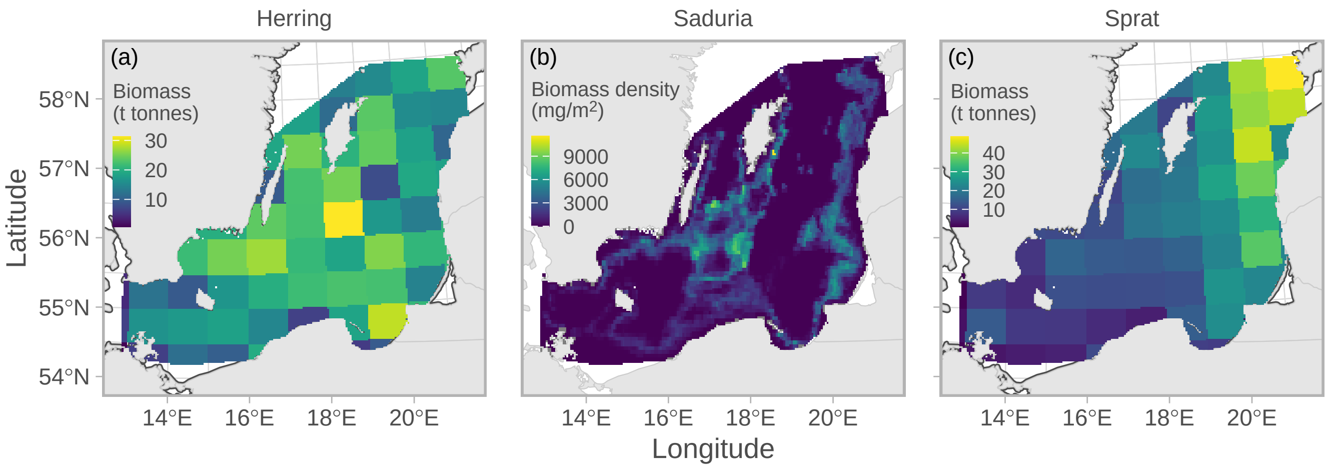

Figure S8: Prey biomass (sprat and herring) and biomass density (*Saduria*) averaged over time (1993–2023).

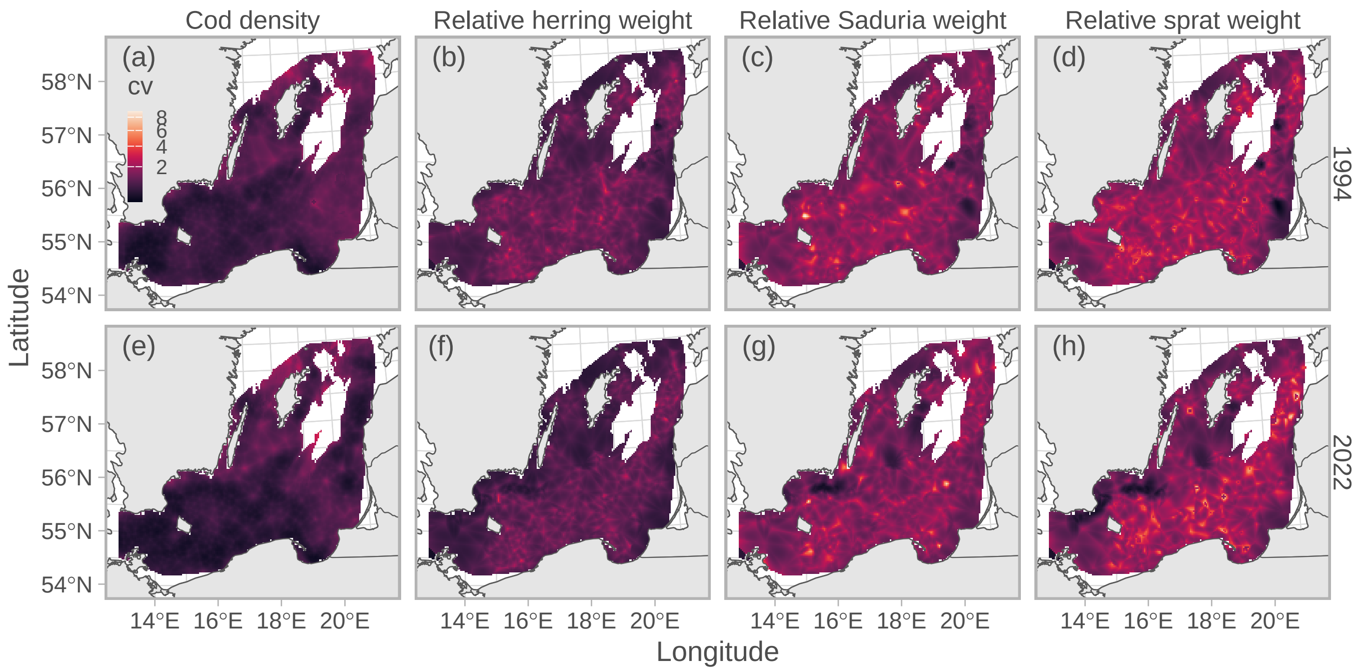

Figure S9: Coefficient of variation (CV) calculated on a grid-cell level across 500 simulations in 1994 (top row, a–d) and 2022 (bottom row, e–h), for predicted cod biomass density (a, e) and relative prey weight of herring (b, f), *Saduria* (c, g) and sprat (d, h). Note that the color scale is square-root transformed. Only grid cells with depth $<$130 m are included in the plot.

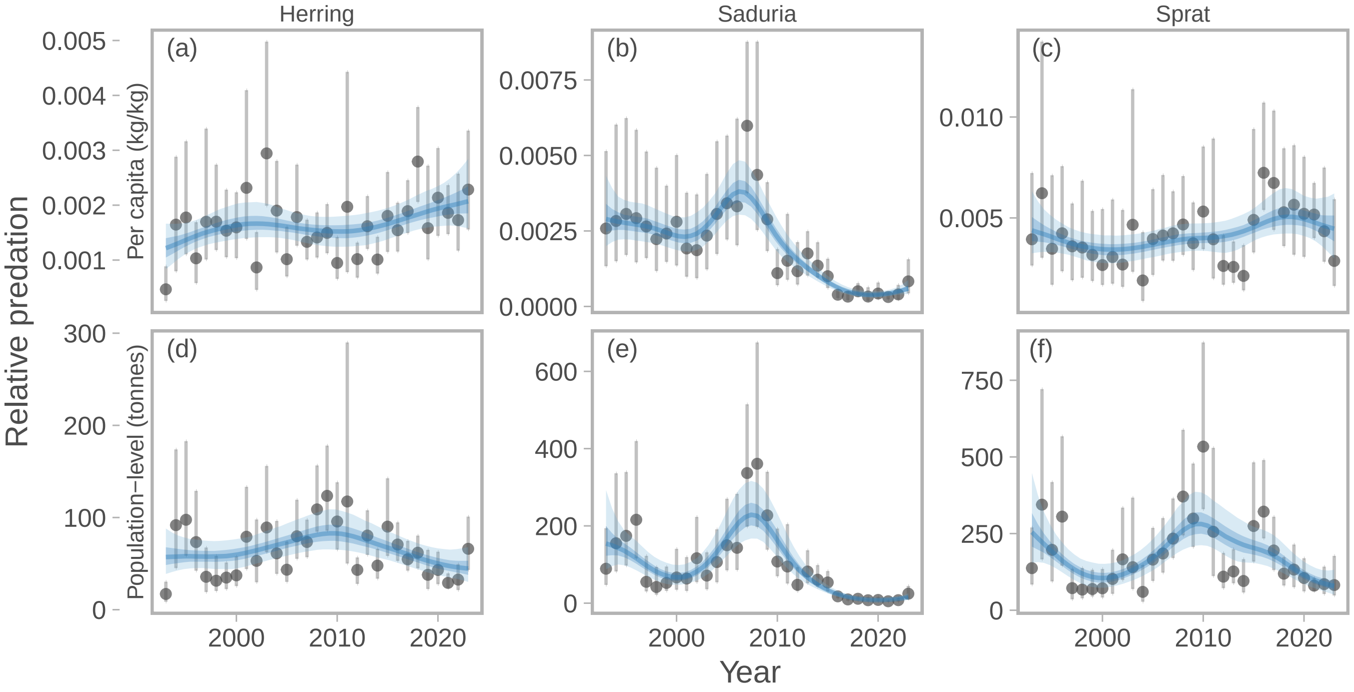

Figure S10: Relative per-capita (top row, a–c) and population-level predation (bottom row, d–f) by cod on herring (a, d), *Saduria* (b, e), and sprat (c, f) over time, *with empty stomachs omitted from the diet models*. Points depict the median predation, and vertical lines depict the range between the 10th and 90th percentile of predation, calculated from 500 simulated spatial predictions of both relative prey weight and cod density. Blue lines depict fits from a generalized additive model with year modelled as a penalized spline, and ribbons correspond to the 50% and 90% credible interval of the prediction.

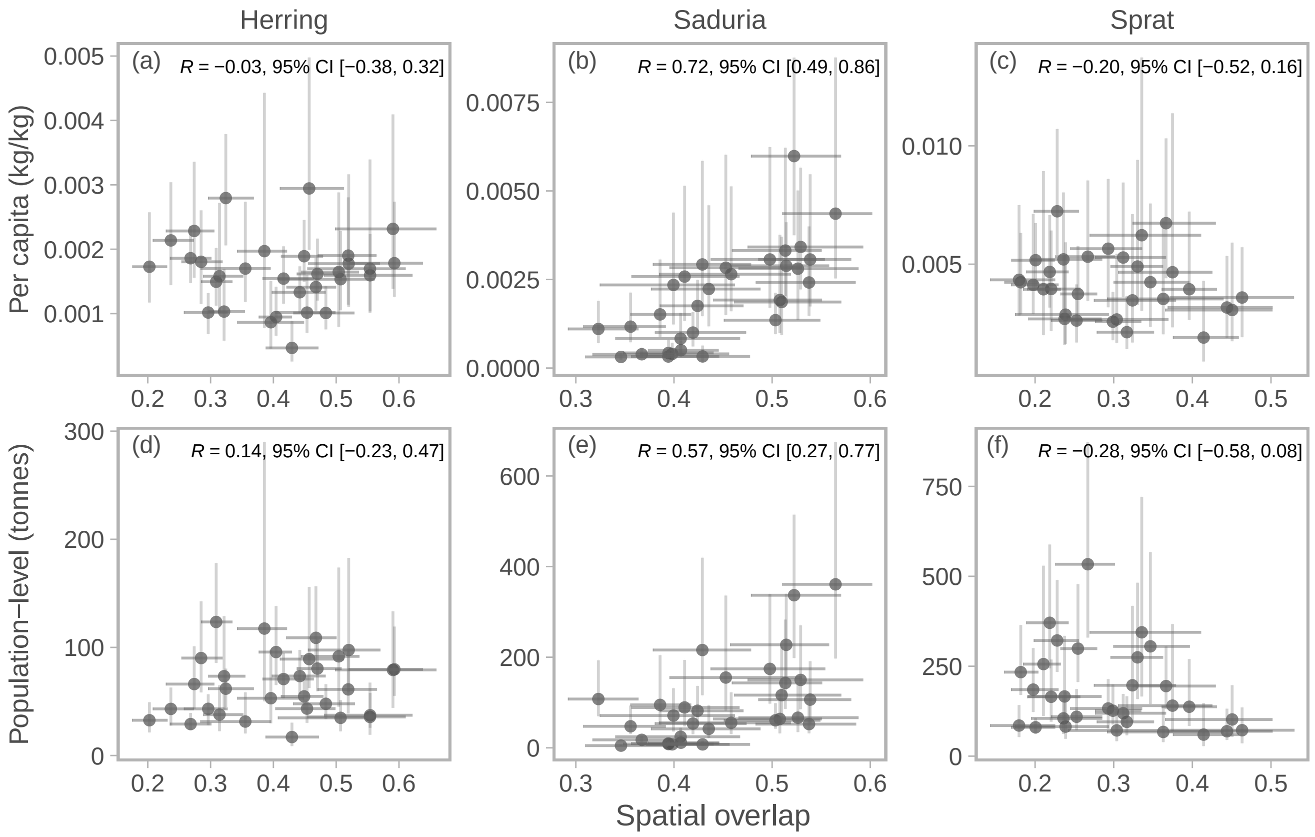

Figure S11: Correlation between relative per-capita predation and spatial overlap (top row, a–c), and relative population-level predation and spatial overlap (bottom row, d–f), *with empty stomachs omitted from the diet models*. Points depict the median, and vertical and horizontal lines depict the range between the 10th and 90th percentile of predation and spatial overlap, respectively, calculated from 500 simulated spatial predictions of predation and cod density. The Pearson correlation coefficient and its 95% confidence interval is printed in the top right corner of each panel for herring (a,d), *Saduria* (b, e), and sprat (c,f).

References

Dunn, P. K., & Smyth, G. K. (1996). Randomized Quantile Residuals. Journal of Computational and Graphical Statistics, 5(3), 236–244. https://doi.org/10.2307/1390802

Hartig, F. (2022) DHARMa: Residual Diagnostics for Hierarchical (Multi-Level / Mixed) Regression Models. URL https://CRAN.R-project.org/package=DHARMa. R package version 0.4.6.
